# Determinants of Adherence to Best Practice in Severe Traumatic Brain Injury: A Qualitative Study

**DOI:** 10.1007/s12028-022-01551-x

**Published:** 2022-08-10

**Authors:** Dashiell Gantner, D. Jamie Cooper, Simon Finfer, Peter Bragge

**Affiliations:** 1 Australian and New Zealand Intensive Care Research Centre, Monash University, Level 3, 553 St Kilda Road, Melbourne, VIC 3004 Australia; 2 Department of Intensive Care, Alfred Health, Melbourne, Australia; 3 The George Institute for Global Health, University of New South Wales, Level 5, 1 King Street, Newtown, Sydney, NSW 2042 Australia; 4 School of Public Health, Imperial College London, London, UK; 5 BehaviourWorks Australia, Monash Sustainable Development Institute, Monash University, 8 Scenic Boulevard, Clayton Campus, Melbourne, VIC 3800 Australia

**Keywords:** Traumatic brain injury, Clinical decision-making, Qualitative exploration, Behavioral determinants, Theoretical domains framework

## Abstract

**Background:**

Management of patients with severe traumatic brain injury (sTBI) is highly variable and inconsistently aligned with evidence derived from high-quality trials, including those examining intravenous fluid resuscitation and use of decompressive craniectomy surgery. This study explored the barriers and facilitators of general and specific evidence-based practices in sTBI from the perspectives of stakeholder clinicians.

**Methods:**

This was a qualitative study of semistructured interviews conducted with specialist clinicians responsible for acute care of patients with sTBI. Interview analysis was guided by the Theoretical domains framework (TDF), and key themes were mapped to relevant TDF behavioral domains.

**Results:**

Ten neurosurgeons, 12 intensive care specialists, and three trauma physicians from six high-income countries participated between May 2020 and May 2021. Key TDF domains were environmental context and resources, social influences, and beliefs about consequences. Evidence-aligned management of patients with sTBI is perceived to be facilitated by admission to academic research-oriented hospitals, development of local practice protocols, and interdisciplinary collaboration. Determinants of specific practices varied and included health policy change for fluid resuscitation and development of patient-centered goals for surgical decision-making.

**Conclusions:**

In choosing interventions for patients with sTBI, clinicians integrate local environmental, social, professional, and emotional influences with evidence and associated clinical practice guideline recommendations. This study highlights determinants of evidence-based practice that may inform implementation efforts and thereby improve outcomes for patients with sTBI.

**Supplementary Information:**

The online version contains supplementary material available at 10.1007/s12028-022-01551-x.

## Introduction

Traumatic brain injury (TBI) is a global health problem: the worldwide annual incidence is 69 million, of which 5.48 million cases are severe TBI (sTBI) [1, 2]. Outcomes following sTBI remain poor: a minority of patients survive to live independently or return to work, whereas more than 50% die or suffer long-term severe disability [3]. Responsibility for the complex acute care of patients with sTBI is frequently shared between specialist doctors from neurosurgery, intensive care, and trauma disciplines. As in many disciplines of medicine, management of TBI varies [4–6], despite international efforts to harmonize management [7–10].

A higher quality of clinical evidence has been associated with increased likelihood of implementation [11, 12]. Of the few sTBI practices tested in high-quality clinical trials [13], two were shown to worsen outcomes compared with alternative treatments, and the results informed clinical practice guideline (CPG) recommendations against their use. First, intravenous fluid resuscitation with albumin increased mortality in patients with TBI admitted to intensive care units (ICUs) [14], informing recommendations that “solutions other than albumin be used in patients with head injury” [10, 15, 16]. Second, for a specific group of patients with sTBI with diffuse brain injury (generalized brain injury and no evaluable traumatic hemorrhages), surgical removal of part of the skull (decompressive craniectomy) can reduce pressure in the skull and reduce days in the ICU, but it increases the number of severely disabled survivors compared with nonsurgical management [17, 18]. This finding underpinned recommendations, such as one by the Brain Trauma Foundation (BTF) that states that “decompressive craniectomy is not recommended to improve [functional] outcomes at 6 months post-injury in severe TBI patients with diffuse injury” [7].

The determinants of evidence-adherent practice in acute sTBI, and of the above practice behaviors specifically, have not been explored from the perspective of treating clinicians. Such enquiry is important to identify factors that determine practice variability and is an essential precursor to developing active implementation strategies aimed at reducing evidence–practice gaps [19, 20]. Furthermore, theory-based analysis grounded in behavioral psychology can aid in the identification and understanding of behaviors that drive practice [21].

This study aimed to identify factors that influence the adherence to evidence in the interdisciplinary acute management of patients with sTBI, with focus on choice of resuscitation fluids and decisions to perform surgery to treat intracranial hypertension, from the perspective of medical practitioners responsible for sTBI care.

## Methods

### Study Design

This was a qualitative study using in-depth semistructured interviews. Approval was obtained from the Monash University Human Research Ethics Committee (project number 23614). This report adheres to the Standards for Reporting Qualitative Research [22].

### Participants and Recruitment

The investigators were intensive care clinician researchers (DG, DJC, and SF), with experience in the clinical management of TBI, and a knowledge translation researcher (PB). All investigators had research experience in international clinical studies in neurotrauma and critical care. Interview participants were hospital specialist clinicians responsible for the acute hospital care of patients with sTBI. During their stay in the ICU, clinical decisions regarding patients with sTBI are usually made by intensive care specialists and neurosurgeons, with input from trauma specialists. Therefore, clinicians from these disciplines were identified using purposive sampling from the professional networks of the investigators. Geography and academic affiliation may influence uptake of evidence; we aimed to recruit clinicians from Australian and international hospitals with comprehensive neurotrauma services.

### Sample Size

Consistent with a qualitative research paradigm, sample size was not predetermined. Sampling continued until no new qualitative themes emerged from three consecutive interviews (qualitative saturation).

### Procedure

Participants received an invitation and explanatory statement by email and were asked if they were willing to participate in an interview lasting 30 to 45 min. Agreement to participate and acknowledgment of the explanatory statement implied consent. Single one-on-one interviews were conducted by the lead author (DG) via videoconference (Zoom Video Communications, Inc., San Jose, CA) with all but two participants, who requested in-person interviews. Interviews were recorded and transcribed verbatim using professional transcription services (Rev, San Francisco, CA). Checked transcripts were imported into NVIVO 12 (QSR International Pty Ltd, Doncaster, Australia) to manage data and facilitate analysis.

### Interview Content

Initial open interview questions explored clinicians’ perceptions of the general determinants of evidence-based practice in sTBI, with focused prompt questions to explore the contribution of CPGs and hospital protocols (see interview guide in Supplementary Materials). Interview content then shifted to the two specific practices shown in randomized controlled trials to alter patient-centered outcomes: namely, fluid resuscitation and decompressive craniectomy for intracranial hypertension. Follow-up prompt questions explored the influence of key clinical trials pertaining to these interventions. The final area explored interdisciplinary dynamics and potential sources of bias between professional groups. The interview guide was developed by investigators with expertise in clinical content (DG and DJC) and qualitative and knowledge translation research (PB). Questions were piloted with two clinical staff, and the interview framework was updated.

### Analysis

Consistent with previous published approaches [23], in this study, the Theoretical Domains Framework (TDF) was used to guide interview coding and analysis rather than to develop the interview guide. The TDF integrates numerous behavioral change theories and consists of 14 domains (groups of constructs from theories of behavior change) that cover factors influencing individual practitioner behavior and behavior change (see Supplementary Table S3) [23, 24].

Using NVIVO®, one investigator (DG) performed open thematic coding and recoding of transcripts in an iterative back-and-forth process. Statements by different participants with similar or related underlying ideas (including statements expressing the opposite belief regarding influence of the same factor) were collected in thematic groups within relevant TDF domains. For example, statements regarding the influence on practice of hospital protocols would be grouped under a theme of ‘Local resources’ within the ‘Environmental context and resources’ domain (Supplementary Tables S1–S3). Emergent themes were subsequently tested for applicability, validity, and consistency. Themes coded to more than one domain were cross-indexed. After three interviews were coded, coding was reviewed with a second investigator (PB).

A domain was considered salient if associated themes were identified consistently by participants or were deemed of high impact on clinical practice by researchers or participants, or if there was a combination of these features.

## Results

### Participants

Between April 2020 and May 2021, twenty-five medical specialists participated in semistructured interviews: ten neurosurgical specialists, 12 intensive care specialists, and three trauma specialists. Participants worked at eight different hospitals that receive and manage patients with sTBI in six high-income countries. All but one hospital had comprehensive neurosurgical services on-site. Participants were predominantly male (84%) and White (80%), of variable age (76% aged 36–55), and working in metropolitan areas (92%). Most were from Australia (76%), and half were from a single academic center (Table 1). Academic appointments were held by 64%, and 56% had worked at least 10 years as qualified specialists.

**Table 1:** Characteristics of sites and participants

Hospital	Region	Metropolitan/regional	Size (large, ≥ 50 admissions/year; small < 50 admissions/year)	Hospital academic affiliation	Neurosurgeon participants	ICU specialist participants	Trauma specialist participants	Total participants
1	Australia	Metropolitan	Large	Yes	4	6	3	13
2	Australia	Metropolitan	Large	Yes	1	1	0	2
3	Australia	Metropolitan	Small	Yes	1	1	0	2
4	Australia	Regional	Small	No	0	1	0	1
5	Australia	Metropolitan	Small	Yes	0	1	0	1
6	European Union	Metropolitan	Small	Yes	0	1	0	1
7	European Union	Metropolitan	Large	Yes	0	1	0	1
8	North America	Metropolitan	Large	Yes	1	0	0	1
9	North America	Regional	Large	Yes	1	0	0	1
10	European Union	Metropolitan	Large	Yes	1	0	0	1
11	European Union	Metropolitan	Large	Yes	1	0	0	1

All sites except hospital 5 were designated major (level I) trauma centers

*ICU* intensive care unit

Each of the practice areas discussed had their own pattern of influencing factors (Supplementary Tables S1–S3). The following is a summary of findings. Illustrative quotations from the transcripts are shown in Table 2.

**Table 2:** Determinants of evidence-based practice in severe TBI

General determinants of severe TBI management	
TDF domain and associated themes	Illustrative quotes^a^
*Environmental context and resources*	
Academic institutional resources	“In a quaternary academic center, there is a focus on academic pursuit and evidence-based practice, and there’s regular review, there’s ongoing peer review, there’s ongoing audit, there's ongoing quality improvement initiatives, people are primed.” (IC01)
Practice audits	“The first thing we did was a survey…to figure out what [TBI] practice was, [and] it was terrible.” (NC02)
Local engagement with research	“It’s certainly an enabler, the degree to which a department is engaged in the research process, broadly and specifically, with respect to TBI.” (IC08)
Policy environment	“There’s an awful lot of patients in the USA that are getting [guideline-adherent care] only because it’s essentially a requirement to be a level one trauma center.” (NC06)
Availability of interventions	“Making it harder to provide an intervention that might not be recommended by the group…certainly would be more useful than writing a lot of guidelines that people may not consult.” (IC07)
*Social influences*	
Collaborative decision-making	“I like talking to my colleagues and talking things over. They have expert knowledge that I don’t have.… A shared model [of care] is undoubtedly the best.” (IC08)
Organizational culture	“I think there needs to be a mutual respect as well as a partnership for what each specialty can offer in terms of advice, opinion, and direction of management.” (NC03)
*Beliefs about consequences*	
Nihilism	“All the interventions we’ve had over the past 20 years, whether it’s hypothermia, decompressive craniectomy, they’re all dangerous processes, and they don’t make you better.” (NC04)
Alignment of metrics	“We always need to identify the issues that can improve our KPIs because we no longer are just looking at survival.” (NC09)
*Behavioral regulation*	
Countering dogma	“I suppose they just had done it their way for so long…they would still be doing it, whatever the trial showed.” (NC03) “I’m not going to use it unless I have some idea…of how it works.” (NC02)
Ease of implementation	“The more complicated TBI interventions are more challenging from that point of view to translate because of the implementation education resources that are required, or the effort that’s involved to change practice.” (IC01)
*Social/professional role and identity*	
TBI not a popular subspecialty	“Traumatic brain injury is not really seen as a route to academic promotion, publications, it’s just not seen as a sexy type of thing for a young and upcoming surgeon.” (NC05)
*Knowledge*	
Mixed perspectives on CPGs	“I think everybody has that experience where you read a guideline and then you actually look up the reference, either discover that the paper that that sentence is based on is very weak or it doesn’t seem to have anything relevant.” (IC05) “It’s really easy to find a reason to do something different.…It’s very rare you have a perfect patient that is described in a perfectly designed trial.” (IC11) “The translation of research findings in the guidelines is very haphazard…it can be years and years and years before [a new finding is] adopted, and incorporated into practice.” (IC04)

Determinants of intravenous fluid resuscitation practice	
TDF domain and associated themes	Illustrative quotes^a^
*Environmental context and resources*	
Policy environment	“Probably the most effective facilitator of the implementation of that particular piece of evidence was now a requirement, where the blood bank will only release albumin when there’s a consent to the administration of blood products.” (IC03)
*Social influences*	
Key opinion leaders	“Getting this information from people like [local champion], huge mentor early on, quickly changed practice. I certainly haven’t prescribed albumin since.” (TC03)
*Knowledge*	
Awareness of evidence	“I think that there was a study from long time ago, I have to look it up, that looked at colloid versus crystalloid.” (NC09)

Determinants of decompressive craniectomy practice	
TDF domain and associated themes	Illustrative quotes^a^
*Beliefs about consequences*	
Craniectomy improves ICP but not long-term outcomes	“If it’s an older patient, who’s got a lot of comorbidity then it’s…not really an appropriate intervention.” (IC01) “If you perform a craniectomy on day two and it gets the patient out of ICU on day five.… We’re looking at perhaps an intervention that’s going to help us in the short-term but may not help in the long-term.” (NC01)
*Social influences*	
Cultural context	“We are in a Catholic culture, so only God can take off your life. This is our culture. So it’s difficult to discuss with family because whenever you say I have 1% of possibility, they want 1%.” (NC07)
*Emotion*	
Emotional pressure	“It’s just really hard if you’re looking at a patient and a family and you know that if you don’t do the decompression, the patient is probably going to die, it’s very hard not to do it even if you know that [the evidence] is suggesting there’s going to be a bad outcome. It’s hard to withhold life-saving care, and I think that motivates an awful lot of us.” (NC06)
*Social/professional role and identity*	
TBI is becoming an ICU specialty	“Whenever we really [appropriately manage] in ICU our patients, the number of decompressions is reduced to a minimum.… Our intensivists don’t leave us many cases with intracranial pressures persistently over 25.” (NC07) “We’ve gone from a very active approach, to much more passive one.” (N01) “In Europe…the primary responsibility for the decision-making remains with the intensivist.” (NC10)

*CPG* clinical practice guideline, *IC* intensive care consultant, *ICP* intracranial pressure, *ICU* intensive care unit, KPI, xxx, *NC* neurosurgery consultant, *TBI* traumatic brain injury, *TC* trauma consultant, *TDF* Theoretical Domains Framework

^a^Some quotations have been edited for readability, including addition of text in brackets, without alteration to meaning

### General Determinants of sTBI Management

The key general factors thought to influence clinical practice in management of patients with sTBI were grouped within six theoretical domains (Supplementary Table S1).

#### TDF Domain: Environmental Context and Resources

#### Academic Institutional Resources, Protocols, and Procedures

On the basis of reported experience and observations, participants from all disciplines stated that practice was more likely to be evidence aligned in academic university-affiliated hospitals than nonacademic hospitals. Hospital characteristics cited as facilitators included presence of subspecialized staff, established internal processes of interdisciplinary engagement and quality improvement and engagement with clinical trial networks.

All clinicians perceived local institutional practice protocols to facilitate consistent evidence-based practice and in some cases, more so than widely disseminated CPGs, such as those produced by the BTF. Others cited the benefit of local protocols in providing pragmatic evidence-based guidance to nonspecialist bedside staff without requiring them to have in-depth knowledge of TBI literature.

Clinicians of all disciplines noted that audit of practice to identify any evidence–practice gaps was an essential prerequisite to motivating improvement.

#### Local Engagement with Evidence Development

Several clinicians felt strongly that local participation in clinical research was associated with implementation of evidence; they reasoned that the interventions tested at their institutions would be relevant to the local patient case-mix, championed by local investigators, and, therefore, readily adopted. Additionally, local research engagement reflects attention to contemporary evidence even beyond those interventions investigated in locally championed studies.

#### Policy Environment and Availability of Interventions

Accreditation by specialist bodies and changes in government policy were highlighted as potent enablers of evidence-based practice. Participants in North America stated that linking accreditation of trauma hospitals with specific elements of the BTF guidelines had improved practice.

Several intensive care clinicians noted that harmful medications and procedures may remain available despite recommendations against their use and that it should be relatively straightforward to make them less available.

### TDF Domain: Social Influences

Social influences related to interprofessional relationships were highlighted by most participants as important determinants of practice. These were frequently cited in association with hospital and environmental characteristics.

#### Organizational Culture and Key Opinion Leaders

Many clinicians favorably cited collaborative, interdisciplinary activities (research, education, protocol development) and management of critically ill patients with TBI as a potent enabler of evidence-aligned care, noting that different specialty groups brought different knowledge and skills. Several participants noted that such collaboration was dependent on a culture of positive, respectful engagement. Advocacy of key opinion leaders was also acknowledged as a driver of practice change and adoption of evidence.

### TDF Domain: Beliefs About Consequences

Multiple participants expressed nihilism about currently available treatments, stating that research has not improved outcomes for patients following TBI and that the best approach is to avoid causing additional harm with potentially dangerous or overly invasive interventions.

Others cited anecdotes of satisfactory patient outcomes following non-evidence-aligned practice to justify such practice or highlight persistent knowledge gaps. Improvements in commonly used but short-term metrics, such as normalization of intracranial pressure or reduced length of stay in ICU, were also cited as possible perverse incentives for non-evidence-aligned therapies. To counter this, performance and audit metrics should align with patient-centered outcomes that include long-term functional recovery rather than short-term physiological markers or surrogate end points.

### TDF Domain: Behavioral Regulation

#### New Versus Old: Countering Dogma

Participants from all specialties opined that current practice, particularly neurosurgical practice, can be dogmatic and based on outdated evidence. Clinicians perceived themselves and others to be averse to change, particularly in situations in which new knowledge conflicted with established paradigms. Treatments were more likely to be adopted if they fitted an accepted model of TBI pathophysiology compared with treatments with mechanisms that were less understood but were nevertheless proven to change outcomes.

#### Ease of Implementation

The simplicity and ease of implementation of interventions tested in clinical trials was perceived to be associated with their rapid adoption, and vice versa. Clinicians noted that this could also be a barrier to evidence-based care, wherein ready availability of harmful treatments enables their inappropriate use. These observations overlapped with themes mapped to environmental context and resources.

### TDF Domain: Social/Professional Role and Identity

Neurosurgeons and ICU and trauma specialists perceived different but overlapping areas of responsibilities for patient care: neurosurgeons did not participate in early in-hospital resuscitation of patients, including fluid resuscitation, whereas ICU specialists perceived that intracranial procedures were exclusively the responsibility of neurosurgeons. All groups felt shared responsibility for overall quality of patient care.

Several neurosurgeons and one ICU specialist observed that most neurosurgeons did not subspecialize in management of TBI, preferring to concentrate on other areas. However, neurosurgeons who delegated responsibility for patient management were perceived to be a potential barrier to evidence-aligned care.

### TDF Domain: Knowledge

Nearly all participating clinicians reported and/or demonstrated knowledge of the pathophysiology of TBI sufficient to appreciate the rationale for different CPGs and usually referred, by name, to the BTF guidelines.

#### Mixed Perspectives on CPGs

However, these CPGs were not consistently held to be facilitators of optimal practice. Several clinicians perceived available CPGs to be inadequate to facilitate best practice, and many presented reasoned judgments for why they may be barriers to evidence-based practice.

The underlying evidence was held to be insufficiently robust or poorly generalizable because of methodological concerns, therefore justifying alternative management. The variable pathophysiology and complexity of TBI was cited as an indication for personalized care on the basis of an individual patient’s characteristics rather than strict adherence to evidence-based guidelines. Some noted the delays between publication of the evidence and subsequent CPGs as a cause for persistent non-evidence-based practice. Finally, the BTF guidelines were held to be rigorously evidence focused at the expense of being nonpragmatic, for example, in not providing stepwise guidance on management of intracranial hypertension because of a lack of high-quality comparative data.

Others held more favorable views on the CPGs as facilitators of evidence-based practice, citing their value in providing instructions for clinicians without in-depth knowledge of underlying science and clinical trial evidence. Additionally, guidelines were held to be a baseline standard against which actual practice could be audited and deviations interrogated.

### Determinants of Intravenous Fluid Resuscitation Practice

Following discussion of determinants of neurotrauma practice in general, interview questions focused on the two treatments shown to be potentially harmful: fluid resuscitation with albumin and surgical decompressive craniectomy.

Key factors reported to influence the choice of intravenous resuscitation fluids following sTBI were grouped within the following theoretical domains (Supplementary Table S2).

#### TDF Domain: Environmental Context and Resources

Restricting availability of potentially harmful fluids was seen as being the most effective mechanism of preventing their administration and rapidly improving practice. Such restriction was reported to have been achieved in some hospitals by charging a high price per unit and by a change to blood bank policies to require consent for administration of blood-derived products, including albumin. Reduced availability would reduce the risk of unknowing clinicians inadvertently prescribing albumin.

#### TDF Domain: Beliefs About Consequences

With few exceptions, in response to open questions on determinants of intravenous fluid choice, participants of all disciplines cited the physiological impact of different intravenous fluid types rather than impacts on patient-centered end points of clinical trials, such as survival or functional recovery.

#### TDF Domain: Knowledge

Participants demonstrated or expressed awareness of evidence regarding the use of albumin that was in keeping with their scope of practice. In practice, neurosurgeons are not involved in fluid resuscitation of patients, and several acknowledged this was outside their knowledge base. Among ICU specialists, several worked in countries where albumin is not readily available, so they reasoned they had little motivation for exploring this evidence. However, some participants in countries where albumin is available were similarly unaware or noted that albumin was not used frequently but were uncertain why. There was greater awareness among intensive care clinicians with clinical research experience or academic appointments.

### Determinants of Decompressive Craniectomy Practice

Key factors thought to influence the practice of using decompressive craniectomy to reduce intracranial pressure were grouped within the following theoretical domains (Supplementary Table S3).

#### TDF Domain: Beliefs About Consequences

Participants’ responses regarding decompressive craniectomy were commonly informed by synthesizing the results of key trials. Clinicians commonly inferred that decompressive craniectomy is an intervention of last resort, only considered after the full range of nonsurgical therapies have failed. Participants held that decompressive craniectomy improves intracranial pressure while acknowledging that it may also increase the number of severely disabled survivors. For this reason, it was felt that use of decompressive craniectomy should be reserved for those with the best chance of neurological rehabilitation: generally younger people and those without preceding functional impairments. Some clinicians emphasized reduction of intracranial pressure (a widely accepted surrogate marker of brain injury and treatment target) as the reason to perform decompressive craniectomy, irrespective of high-quality evidence showing harm.

#### TDF Domain: Social Influences

#### Cultural Context

Cultural context was noted to be an important factor in deciding whether to perform life-sustaining surgeries with the expectation that the patient may survive with debilitating disability. Clinicians noted that within some ethnic or religious communities, human life is considered sacred in any condition, meaning there is a default to active intervention even in situations in which clinicians might consider the prognosis unfavorable.

#### TDF Domain: Emotion

Despite being cognizant of the risk of poor outcomes, participants from all disciplines (particularly neurosurgeons) reported emotional pressure to perform decompressive craniectomy surgery when presented with a patient with refractory intracranial hypertension. This pressure was both external (from families or other clinicians) and internal (a human bias toward active intervention).

With few exceptions, discussions with relatives or friends of the patient to determine their values were reported to be essential in deciding therapies. If a family expressed the view that a patient would not wish to survive with severe disability, this may prompt clinicians to advocate for less invasive (nonsurgical) therapies. Some clinicians noted that families may struggle to incorporate the complexity and uncertainty of management and may advocate for decompressive craniectomy to be performed in the belief that the more invasive therapy is more likely to result in improved outcome.

#### TDF Domain: Social/Professional Role and Identity

Given the complexity of decision-making, clinicians from all groups reiterated that collaborative interdisciplinary discussions at a senior level were essential to decide the most appropriate management for individual patients. Although decompressive craniectomy can only be performed by neurosurgeons, both they and ICU specialists perceived that they share responsibility for decisions regarding surgery. In some regions this has shifted over time such that greater responsibility now rests with the ICU. One academic neurosurgeon noted favorably that few patients have refractory intracranial hypertension if nonoperative therapies are used appropriately; therefore, decompressive craniectomy is rarely indicated and the evidence is rarely applicable.

## Discussion

In this qualitative exploration of stakeholder clinicians’ perceptions of the acute management of sTBI, we found that in determining practice, evidence-based recommendations are integrated with local environmental, social, professional, and emotional influences. Our study builds on previous findings that adherence of neurocritical care practice to evidence is a function of factors related to institutions, clinicians, and the guidelines themselves [12, 25, 26]. Key domains of the TDF were identified, including environmental context and resources, social influences, and beliefs about consequences, highlighting the utility of this framework in analysis of knowledge translation. When evidence–practice gaps exist in sTBI, implementation requires attention, tailored for different practices, to each of these areas.

The health care environment is a potent facilitator of evidence-based sTBI practice in general through different mechanisms at different levels (from policy environment to hospital resources) depending on the practice behavior. Among the sampled clinicians, organizational culture promoting interdisciplinary academic activities (education of trainees, participation in clinical research, regular practice audits, and local treatment protocols) and presence of key opinion leaders were consistently cited as facilitators of evidence-based practice, and interdisciplinary conflict arising from inadequate collaboration was seen to be a barrier. These findings are consistent with previous studies of clinicians’ perceptions of enablers of guideline-based acute hospital care in neurotrauma and antenatal settings [25, 27] and with our previous study showing close alignment between practice and evidence in academic neurotrauma centers in Australia [28]. Our findings support promotion of academic interdisciplinary collaboration to promote shared understanding of the evidence and enable standardization of practice, such as has been achieved in other critical care settings [25, 29, 30]. Allocation of hospital resources to development of pragmatic, local, interdisciplinary management protocols based on CPGs is perceived favorably and can improve outcomes [31]. Such collaboration should encourage improved interaction between specialist groups, assist in clarifying roles and responsibilities, and minimize barriers associated with the behavioral domain of social/professional role and identity.

Evidence-based fluid resuscitation practice in TBI can be achieved with changes to the policy environment. Restricting availability of albumin is relatively uncomplicated given that safer alternatives exist with fewer barriers to administration. Fluid resuscitation practice in critically ill patients has changed in response to evidence [32], but in previous work, we showed that in settings where albumin remains available, some patients with sTBI will continue to receive it inappropriately [28].

Enabling evidence-aligned use of decompressive craniectomy is more complex, with influential factors intersecting multiple TDF domains. A decision whether to offer decompressive craniectomy is usually made at a time of emotional stress for families and clinicians, when nonoperative treatment has failed to treat intracranial hypertension and patients are at immediate risk of dying. Few participants explicitly prioritized high-quality clinical evidence comparing patient-centered end points over lower-quality evidence. Although participants demonstrated knowledge within expectations for their specialty groups, at times their awareness of established models of TBI pathophysiology and the likely physiological impacts of specific therapies overrode or appeared to compete with adherence to evidence-based CPG recommendations. Consistent with previous findings, this was particularly true if the mechanisms of the recommended intervention were inconsistent with the clinician’s existing knowledge [25]. Clinicians may consider CPGs as just one of multiple influential factors, which prominently include short-term surrogate measures such as intracranial pressure. Our findings suggest the need for clinicians to explicitly prioritize CPG recommendations aligned with patient-centered outcomes (survival and long-term functional recovery) over short-term and potentially misaligned pathophysiological end points (such as intracranial pressure). Notably, evidence-based CPGs, including those produced by the BTF, do not refer to patient-centered decision-making, and complex deliberations of death versus survival with disability may be deferred to families, causing significant psychological distress [33]. In this context, the assessment of adherence to evidence to practice is nuanced: decisions on invasive treatments should be made as part of a management pathway that prioritizes individualized long-term goals but is informed by short- and medium-term physiological targets (in which high-quality evidence may be lacking). Our findings support the routine training of neurotrauma clinicians in models of shared decision-making to ensure appropriate incorporation of individual patients’ values and patient-centered goals [34, 35].

Previous studies suggest that adherence to CPGs is enhanced when clinicians have confidence that the recommendation will produce the desired outcome [36]. Nearly all participants in our study had knowledge of the evidence base sufficient to critique or disregard the CPGs (including a recent BTF update on decompressive craniectomy) [37], in some cases providing a reasoned disagreement on their interpretation or justification for why their practice was not adherent, citing methodological limitations of studies or the complex pathophysiology of sTBI. A dominant theme in these participants’ responses was the inability of trials or associated CPGs alone to provide sufficient guidance to individualize care. This may reflect skepticism regarding the historically poor quality of CPGs in critical care [38] and is consistent with previous findings of doctors being knowingly selective in their application of CPGs [25, 39]. This highlights the need for additional high-quality research addressing questions of pragmatic clinical relevance to clinicians treating patients with sTBI.

### Strengths and Limitations

This study has several notable strengths, including sampling of clinicians from multiple specialties involved in the care of patients with sTBI, interviews conducted by a content expert, and analysis of influential factors using a validated theoretical framework by content experts and an experienced knowledge translation researcher. Our study is unique in exploring the interdisciplinary dynamics associated with acute management of sTBI and in examining determinants of two specific practices. Our study also has some limitations. We used the TDF predominantly in the analysis of responses rather than in the development of the interview guide; assignment of responses to TDF domains was post hoc, but this approach allowed for analysis according to dominant, unprompted themes. The clinicians were predominantly sampled from a single Australian academic neurotrauma center, with potential for unconscious or confirmation bias to influence perspectives. Although participants ranged in age and breadth of experience, the factors they identified are relevant to the circumstances in which they were sampled and may not be relevant with changes in practice, environment, and evidence. These issues, as well as the limited racial and gender diversity of respondents, potentially limit the generalizability of our results.

## Conclusions

In a sample of predominantly Australia-based specialist clinicians, factors influencing management decisions and the implementation of key evidence-based practice recommendations for sTBI were identified. Academic organizational culture, resources, and interdisciplinary collaboration were determinants that facilitated evidence-based practice. Fluid resuscitation practice could potentially be improved with health policy changes, whereas an explicit focus on patient-centered treatment goals could direct optimal surgical practice. This study highlights determinants of evidence-based practice that may inform tailored implementation efforts in similar settings and thereby improve outcomes for patients with sTBI.

## Supplementary Information

Below is the link to the electronic supplementary material.

Supplementary file 1. Interview guide for clinicians involved in acute management of severe traumatic brain injury.

Supplementary file 2. Table S1. Factors perceived generally to influence practice in severe traumatic brain injury; Table S2. Factors perceived to influence practice in fluid resuscitation of patients with severe traumatic brain injury; Table S3. Factors perceived to influence practice of surgical decompressive craniectomy in patients with severe traumatic brain injury.
