# Supplementary material for: Determinants of Adherence to Best Practice in Severe Traumatic Brain Injury: A Qualitative Study
Source: Neurocrit Care. 2022 Aug 10;37(3):744–53. doi: 10.1007/s12028-022-01551-x (PMC9672018; doi:10.1007/s12028-022-01551-x)
Supplement: Supplementary file 1 — Interview guide for clinicians involved in acute management of severe traumatic brain injury. [file 12028_2022_1551_MOESM1_ESM.docx]

# Additional file 1:

## Interview guide for clinicians involved in acute management of severe traumatic brain injury

### Introduction

As outlined in the explanatory statement, this study aims to explore factors influencing the management of patients with severe traumatic brain injury.

Using a semi-structured interview framework, I am interviewing clinicians involved in the care of these patients to learn from their experiences, including specialists from different disciplines and departments to get a broad view.

Before we start do you have any questions?

### Background information

- Can you provide a brief introduction and outline your experience in the management of severe traumatic brain injury?

### Management of severe traumatic brain injury (sTBI) – overview of influential factors

- From your perspective what are the factors (the barriers and facilitators) that influence the uptake of TBI research findings and their implementation into clinical practice?
- What strategies are you aware of to minimise the gap between evidence and practice?
- How successful have these strategies been? Why?
- Are you aware of any clinical practice guidelines in this area? What role do guidelines play?

### Specific recommended practices

I’d like to move on to discuss some practices that have been tested in the Australian and New Zealand neurotrauma research community; specifically, choice of intravenous resuscitation fluids and surgical management of intracranial hypertension.

#### Recommended practice: Solutions other than albumin be used in patients with head injury

- What factors influence choice of intravenous fluid type used in patients with traumatic brain injury?
- Are you aware of any clinical practice guidelines (or relevant clinical trials) in this area?
- How do you apply these in your practice?

#### Recommended practice: Bifrontal DC is not recommended to improve outcomes…

- Based on your knowledge and experience, what are the issues to consider when deciding whether to perform craniectomy to reduce intracranial hypertension?
- Are you aware of any clinical practice guidelines or relevant trials in this area?
- How do you apply these in your practice?

### Perceptions and potential sources of bias between professional groups

- How do you view the roles of the Neurosurgery/ICU/Trauma teams in the management of patients with sTBI?

### Closing

- Do you have any other comments regarding evidence-based practice in management of traumatic brain injury?

Thank you for your time today.

Prompt questions to explore factors influencing practice (grouped by TDF domains).

| **TDF Domains** | **TDF Definitions [Constructs]**^1^ | **Prompt questions** |
| --- | --- | --- |
| Knowledge | An awareness of the existence of something. [Knowledge including knowledge of condition/scientific rationale. Procedural knowledge. Knowledge of task environment.] | - What strategies are you aware of to minimise the gap between evidence and practice? - Are you aware of any clinical practice guidelines in this area? What role do guidelines play? |
| Skills | An ability or proficiency acquired through practice. [Skills Skills development Competence Ability Interpersonal skills Practice Skill assessment] | - How easy is it to apply the guidelines in area x? |
| Social/professional role and identity | A coherent set of behaviors and displayed personal qualities of an individual in a social or work setting. [Professional identity Professional role Social identity Identity Professional boundaries Professional confidence Group identity Leadership Organizational commitment] | - How do you perceive the roles of the ICU/Neurosurgery/Trauma teams? - Any differences in skills sets/knowledge? - How do they communicate with families? |
| Beliefs about capabilities | Acceptance of the truth, reality, or validity about an ability, talent, or facility that a person can put to constructive use. [Self-confidence Perceived competence Self-efficacy Perceived behavioral control Beliefs Self-esteem Empowerment Professional confidence] | - How capable do they feel in interpreting evidence? - How confident are they managing patients with sTBI? |
| Beliefs about consequences | Acceptance of the truth, reality, or validity about outcomes of a behaviour in a given situation. [Beliefs Outcome expectancies Characteristics of outcome expectancies Anticipated regret Consequences] | - Does your management change outcomes for patients? - How does [recommended practice x] impact patients? |
| Motivation and goals | A conscious decision to perform a behavior or resolve to act in a certain way. Mental representations of outcomes or end states that an individual wants to achieve. [Stability of intentions Stages of change model Transtheoretical model and stages of change Goals (distal/proximal) Goal priority Goal/target setting Goals (autonomous/controlled) Action planning Implementation intention] | - What motivates clinical decisions in these circumstances? - Are there incentives for [recommended practice]? |
| Memory, attention and decision processes | The ability to retain information, focus selectively on aspects of the environment and choose between two or more alternatives. [Memory Attention Attention control Decision making Cognitive overload/tiredness] | - Do you use any decision aids or flowcharts to help with decision making? |
| Environmental context and resources | Any circumstance of a person’s situation or environment that discourages or encourages the development of skills and abilities, independence, social competence, and adaptive behavior. [Environmental stressors Resources/material resources Organizational culture/climate Salient events/critical incidents Person x environment interaction Barriers and facilitators] | - Does the hospital environment have an effect on practice? - Is your hospital part of a research or academic network? - What educational activities does your hospital support? - Does the availability of resources affect practice? |
| Social influences | Those interpersonal processes that can cause individuals to change their thoughts, feelings, or behaviors. [Social pressure Social norms Group conformity Social comparisons Group norms Social support Power Intergroup conflict Alienation Group identity Modeling] | - Do you seek opinions of colleagues in making these decisions? - How does the shared responsibility for patients influence practice? |
| Emotion | A complex reaction pattern, involving experiential, behavioral, and psychological elements, by which an individual attempts to deal with a personally significant matter or event. [Fear  Anxiety Affect Stress Depression Positive/negative effect Burn-out] | - Is there a human bias towards more active intervention in these cases? |
| Behavioral regulation | Anything aimed at managing or changing objectively observed or measured actions. [Self-monitoring Breaking habit Action planning] | - Are there institutional protocols that help guide practice? |

1. French SD, Green SE, O’Connor DA, et al. Developing theory-informed behaviour change interventions to implement evidence into practice: a systematic approach using the Theoretical Domains Framework. *Implementation science : IS*. 2012;7(1):38. doi:10.1186/1471-2458-8-326
