# Supplementary material for: Determinants of Adherence to Best Practice in Severe Traumatic Brain Injury: A Qualitative Study
Source: Neurocrit Care. 2022 Aug 10;37(3):744–53. doi: 10.1007/s12028-022-01551-x (PMC9672018; doi:10.1007/s12028-022-01551-x)
Supplement: Supplementary file 2 — Table S1. Factors perceived generally to influence practice in severe traumatic brain injury; Table S2. Factors perceived to influence practice in fluid resuscitation of patients with severe traumatic brain injury; Table S3. Factors perceived to influence practice of surgical decompressive craniectomy in patients with severe traumatic brain injury. [file 12028_2022_1551_MOESM2_ESM.docx]

# Additional file 2

## Table S1: Factors perceived generally to influence practice in severe traumatic brain injury

| TDF Domain | Themes | Specialists | Illustrative quotations |
| --- | --- | --- | --- |
| Environmental context and resources | Academic institutional culture and infrastructure associated with evidence-based practice | NC, IC, TC | “It being a teaching hospital, [there is] a constant stream of registrars studying and sitting for exams you need to a certain extent keep up with the guidelines.… I actually find that super helpful, particularly because I’ve had a large number of breaks in practice over the last few years there are large things that I have to wing, so I’m happy for that constant exam practice and supervision.” (IC09)  “There's no doubt that if you're working in a quaternary academic centre, where there is a focus on academic pursuit and evidence-based practice, and there's regular review, there's the ongoing peer review, there's ongoing audit, there's ongoing quality improvement initiatives, people are primed. There's a culture there to allow you to very quickly identify something, take it off the shelf if it's a well-proven product, and slot it into your system. I think that's very true. Whereas if you're working in a smaller place, or in a place that doesn't have that same culture, then yeah, you're going to have to do a lot more work to get people incentivized, to get them invigorated and get them thinking about translating that work.” (IC01)  “I would imagine it would be very easy if someone suffered TBI in a rural centre for a well intentioned doctor, looking after that patient to do things that were perhaps not evidence based. Because their awareness of the evidence may be limited, or they may have worked in academic centre 10 or 15 years earlier. And it's extremely difficult for them to stay up to date with those things. And they'll be in a crisis management mode, and they won't consult any guidelines at that point in time. So I think absolutely in non academic centres, it would be very easy to not perhaps know the best thing to do in the first hours of care.” (IC07)  “[In non-academic centres] there is no multi-disciplinary meeting that is occurring to the detriment, I think, of the patient. Sometimes there's not enough interested people as well on both side who are interested in making that decision. Some devolve to a senior registrar. And sometimes VMO consultants are involved where sometimes VMOs are not that interested as well. The senior registrar is making the decision on very patchy information often.” (NC05)  “It's when you go to a non-academic centre and you try and get them to come onboard with a trial or with new evidence base, then that's much more challenging because the doctor in these hospitals are not attuned to clinical research. They're much more attune to just day-to-day practice, churning through the numbers. "This is how we've always done it, and we can keep doing it because we're happy." Getting them to change practice is much harder.” (NC03)  “I think residents are probably, they're there to learn, they want to do the right thing, and they get a low salary slave labour rate, so they want to do the right thing. So, you get somebody who's very young generally, is more willing to change than somebody's who's very old, and so it's generally true and not totally. So, that's a driver as well, I'd say being an academic institution with residents is more of a driver.” (NC02)  “And then the other facilitator is having groups like the Australian Trauma Society, the leaders that you guys are, ANZICS, and convergence between all these groups, including neurosurgeons, to try and find a solution. There is that research camaraderie, academic camaraderie, and also the agreement that we need to come to a consensus and find solutions for this.” (TC01) |
|  | Local engagement with evidence development is strongly associated with evidence-based practice | NC, IC | “…research that is being generated in a local context, I think has got a much greater penetration to clinical practice in Australia and New Zealand. So I think that a lot of the material or the content is generated by studies outside our system, particularly in North America where they have very different intensive care and neurocritical care set-up. I think it has less translation here, because I think it's considered to be a little remote from us, a bit distant. It doesn't quite fit our practice patterns and it's such, again, a very specialized or sub-specialized area of practice at those other centres… I think if it's homegrown, I think if it's developed by investigators here in Australia that take into consideration the clinical context that we operate in, then I think that those tend to facilitate translation.” (IC01)  “The facilitators first are working in an environment where there are a concentration of clinicians with a strong interest in that area, and probably I'm working in an area where we have clinicians who have contributed to that research or similar research themselves so that they can be champions for its uptake. And perhaps even working in a centre where you as a clinician participated in that research by looking after patients who were randomized into the study in question which is now aiming to change practice.” (IC06)  “I think it's certainly an enabler, the degree to which a department is engaged in the research process, broadly and specifically, with respect to TBI.” (IC08) |
|  | Use of local institutional protocols | NC, IC, TC | “I think the best way is to have all of the different stakeholders sit down together and come up with a local protocol. So something that builds off of all the different relevant guidelines, things that reflect what you have in your own stock room, something tailored to local resources, but a general template for what everyone agrees that they're going to try and do.” (NC06)  “I think that culture [of guideline development] really allows you to translate research, because you can have a greater degree of compliance with a unit-wide protocol… That has its disadvantages, because you may reduce people's individuality a little bit, or initiative or different schools of thought or different approaches. But it allows you to apply a new intervention very uniformly across an organization.” (IC01)  “I think people use guidelines when they really don't know what they're doing, and there's a complex thing they need to solve that they don't think they can answer otherwise.” (IC07)  “I think the local institutional guidelines hugely benefit more than the international ones. I imagine those obviously inform the local guideline development, but the local guideline development has to have buy-in from the players at your institution. Whereas no one's necessarily agreed on the brain trauma foundation guidelines and they're pretty wishy-washy in a lot of places.” (IC08)  “The only difficulty is that, obviously, we have local guidelines, so there does tend to be a lag between what comes out in a research trial and then comes out in the ESICM guidelines and then gets transcribed into our guidelines. That can be a bit of a barrier then, because the nurses will go with the local guideline.” (IC10)  “It's frustrating because you spend time in trying to develop some sort of guidelines and then they're not even followed or nobody even looks at it.” (NC08)  “Whenever I deliver a guidelines here, I need the guidelines for transfer. I need the guidelines for telecommunication. I need guidelines for transfer to rehabilitation, is different. Anyway, the people is very happy to follow reasonable guidelines, because they know that if they do it, they will avoid legal issues. Because I have done this with this patient, but I have followed the best of possible guidelines for my hospital.” (NC07)  “But do we as individual clinicians, are we across everything that we should be of those protocols? No… There's guideline fatigue.” (TC03) |
|  | Local decision support aids | TC | “There's so much stuff that's being written, and to try and integrate it all is very difficult, but I think people are doing a reasonably good job. I think the hospital guidelines are good. I think a lot of that decision support system for the Bonanza study, where you've got some objective support for ... and it's often when you're busy. All of a sudden, you haven't got a drop in perfusion, you're busy doing other things, and you've got something to prompt you, I think that's what we should be doing.” (TC02) |
|  | Policy environment / availability of intervention | NC, TC | “The American College of Surgeons endorsed it, and then they said in their book, every trauma centre, you must follow the Brain Trauma Foundation guidelines, or you're not going to get certified… They're going to be concerned about that, because if they're not a level one trauma centre, they're going to have a problem… and then it went up a lot, but it took 20 years. No, just because you write an article doing a journal in medicine or something, it's not changing. You need to have a little bit of a carrot and stick.” (NC02)  “The other important driver in America is the American College of Surgeons as enforcers. There's an awful lot of patients in the US that are getting ICP monitoring only because it's essentially a requirement to be a level one trauma centre. And if it wasn't for the fact that there's continuous audits and knowledge that the American College of Surgeons is going to come in and ask questions if you don't do it, I think that the system in the US has made some of those practices…the path of least resistance to just do it.” (NC06)  “So we have shown with our data that the surgical decompression, the surgery, meaning that decompression produce improvements, statistically significant, against the medical treatment after one year of management. At six months, as you have seen with data, this is not that statistically significant. It became significant at one year. What does it mean that you have to cure severe head injuries for one year? If you believe we have looked at the social organization, health care, and so on. Only nine countries in the world, only nine allows payment for patient for one year after a head injury. And two are Italy and UK, two of them. So 85% of patients are cured in countries where you cannot export the way in which they are curing patients, because many countries will not pay for that management, including US. And this for me is the key issue. How can we transfer the huge research we are doing in the Western world to the people who is in need?” (NC07)  “It's easy for somebody who comes from a different specialty or from a different part of the hospital, to prescribe the wrong therapy or non-evidence based therapy…I think something like giving albumin to one of these patients is very easy. Whereas, it's not very easy for a patient to go for a decompressive craniectomy. That's not easy, because the consultant will always be called and there's a few more barriers to that happening… making it harder to provide an intervention that might not be recommended by the group, I think certainly would be more useful than writing a lot of guidelines that people may not consult.” (IC07)  “By far the best way to change practice is not trying to change individual practice. It's systems things. So, like telling the clinicians to stop giving albumin. Well, the reason it was given usually is because it's the registrar that prescribes it and he doesn't know. So the way it was dealt with in the end was that blood transfusion [protocols] took albumin out of our unit. And then it was just a pain in the ass to order albumin that it never got prescribed anymore.” (IC08)  “Probably the most effective facilitator of the implementation of that particular piece of evidence was now a requirement, where the blood bank will only release albumin when there's a consent to the administration of blood products.” (IC03) |
|  | Material resource availability | IC | “Anything that kind of had a resource implication it would be a potential barrier, because resources are limited, like even here or at [Hospital] and it's not kind of a resource limited environment like the kind of second or third world.” (IC02)  “I guess the other major thing is cost. I think that there is a financial implication, a lot of the time with some of the interventions, particularly around some of the newer technologies. So, it is a factor, perhaps not, it's obviously not a priority, but it certainly is a factor. I think people perhaps require a higher level of evidence to implement new devices or therapies where there is a cost implication.” (IC01) |
| Social influences | Collaborative, interdisciplinary decisions enable evidence-based practice…  …or inhibit them. | NC, TC, IC | “One of the advantages of the collaboration is not only that you can work with those specialists on the patient group that we're all looking after. Again, we can't do that on our own. But, also, you're broadening it to a larger number of patients and, beyond that, in your own hospital to then multicentre studies and international studies. I think that's been the great spinoff of being able to work with my ICU colleagues, particularly [Investigator] and the team in the [Hospital], who really have pushed the field at the cutting edge as well. Again, they can't really do that without the collaboration and cooperation of people like me who are also in a position to change management practices and develop new protocols, because anything around TBI will involve these multiple specialties, particularly around ED anaesthesia and even the paramedics.” (NC03)  “I think a collaborative approach is important, and I think we should be more collaborative…If we had [a different] model where we were doing everything and calling the neurosurgeons as technicians, I think our patients would be safe and they'd receive good treatment, but I think there's always benefit from us having them on board and us learning from each other.” (IC06)  “I don't want to be sole decision-making, I like sharing that, I like talking to my colleagues and talking things over and I get a lot of feedback from that, and I get a lot of help from that. They have expert knowledge that I don't have. Of course they do. Massive amounts. So I think a shared model is undoubtedly the best” (IC08)  “You need buy in from all the other teams. It doesn’t work if we’re they only ones up to date with the guidelines and journal articles… You need multidisciplinary buy in.” (IC09)  DG: “Do you find that the shared care model is an enabler of evidence-based practice?” IC10: “Honestly, it's worse. Some of that is personality driven, but it leads to… a lack of clarity about who makes the decisions. It can be frustrating, particularly because, as I said, TBI isn't the primary focus of any of the neurosurgeons, so it can be quite difficult to get them to engage.” (IC10) |
|  | Local key opinion leaders change practice | NC, IC | “We do have some thought leaders at [Hospital], like [Specialist] and myself, who are more academically inclines and more attune to the evidence base. Then we'll push that with our colleagues, and get the changes agree to and introduced. That's a process that is working. Could argue that it could be done even more than it is, but it's working.” (NC03)  “It often comes down to individuals, doesn't it? It comes down to champions, it comes down to people that really promote this work.” (IC01)  “I suppose if there was proposed practice change that was promoted or advocated by people like [Specialist] or [Specialist] or [Specialist] that would mean and people would had a high kind of profile within the intensive care communities, people that were the lead kind of experts in critical care, that would carry a lot of weight and people prove more likely to follow that.” (IC02)  “[Specialist] is unequivocally a local champion with respect to implementation of changes in practice with respect to TBI. So he's been a major facilitator.” (IC03)  “The facilitators first are working in an environment where there are a concentration of clinicians with a strong interest in that area, and probably working in an area where we have clinicians who have contributed to that research or similar research themselves so that they can be champions for its uptake.” (IC06) |
|  | Culture of collegiality and respect promotes good practice | NC, IC | “If we want to talk about the management of their ICP, whether their coming to the time of needing surgery, obviously the neurosurgeons are going to be involved. I think there needs to be a mutual respect as well as a partnership for what each specialty can offer in terms of advice, opinion, and direction of management.” (NC03)  “There has to be an environment where there is collegiality and open and nonjudgmental transfer of ideas.” (IC06)  “The degree of collaboration between departments [is important], because TBI research is going to, very rarely, at least for ICU, be an island. It's going to rely on your neurosurgical colleagues and everyone else. If you don't have a collegiate environment, it's going to be hard to agree on stuff and change things. I mean, really, incorporating research and findings into practices about change, being open to change, and having a process where that's looked on favourably.” (IC08) |
| Beliefs about consequences | Trials show active interventions can do more harm than good / nihilism | NC | “All the interventions we've had over the past 20 years, whether it's, hypothermia, decompressive, craniectomy, they're all dangerous disease processes, and they don't make you better. They can stop you dying, but they do not improve your outcome. The brain is very fragile and the more we intervene, the more damage we do. We manipulate the ICP and do all these things, which actually don't improve outcome at all. And they might reduce mortality, but the less you do, actually, the better… We're changing the way we think about TBI now, I think, hopefully. We've gone back from the sort of we're trying to improve outcome by all these therapies by saying, "Okay. Is this person salvageable? Yes or no? Is he going to die? Yes or no?" If he's not going to die and he's salvageable, then don't do anything… in terms of how have we incorporated research, well we have incorporated it, and that's made us stop doing things that we've been doing.” (NC04)  “The barriers is I think human pessimism. That stage where maybe in the last 10 years there have not been major changes for functional outcomes related to any particular study or any particular intervention. There is a pessimism within the neurosurgical community, the intensive care community, and among all types of critical care community about what are going to be the gains?” (TC01) |
|  | Sentinel (anecdotal) experiences trump evidence | NC, IC, TC | “We still have some neurosurgeons who tell us they've had patients they decompressed, who come back and see them in clinic. And so they clearly have their little bias” (IC07)  “And we've seen young people, and young people in particular, who come in, who've been hypotensive for a long period of time, but they seem to wake up fine, and yet some people don't do so well. So we've got these tenants of care, which will be accepted now, those, as appropriately evidentially-based, but they've just become culture and practice.” (TC01) |
|  | Improvements in surrogate outcomes may incentivise non-evidence based care | NC, IC | “I use the example, if you perform a craniectomy on day two and it gets the patient out of ICU on day five that might be what works in your environment, in terms of capacity for supporting long-term ICU patients. We're looking at perhaps an intervention that's going to help us in the short term but may not help in the long term.” (NC01)  “So the ICP may be a useful marker for secondary brain injury. But manipulating it does not help at all.” (NC04) |
|  | Audit metrics should align with evidence-based practice | NC | “We always need to identify the issues that can improve our KPIs because we no longer are just looking at survival. Survival is a poor KPI, right? We're looking at a lot of other things, and we have to look from a patient point of view, a society point of view as well as an institutional and a financial and finally community, I mean national point of view.” (NC09) |
| Behavioural regulation | New evidence may conflict with established knowledge and practice | NC, IC, TC | “I guess we're all by this stage quite set in our ways. So as a practical matter, I guess it's not just knowing the knowledge, but at a practical level being convinced that doing things that way, is actually going to be better than what I've been doing, for the last 10 years kind of thing. So just sort of, I think just changing any sort of habits, developing new habits and practices is a challenge. I think it's just makes our work life easier, they have kind of set, kind of cognitive routines and we work on pattern recognition and we probably implement pattern kind of management for a lot of stuff. (IC02)  “I think my view would be that neurosurgeons in particular often have strong views, and their prior belief, if it's not in accordance with the results of a clinical trial, can cause them to find reasons to disregard the results of the trial.” (IC03)  “I think in Australia we're pretty, really, compared to perhaps other parts of the world, I don't think we have such deep rooted aversion to change, but I don't have to go too many leaps of imagination, to imagine in certain countries or societies where there is over-riding attitudes that are just resistant, because they don't want to shift from an active point of view.” (NC01)  “…the way that surgeons were trained in the past, perhaps not so much now, you were taught in a certain way, and that way has worked and now you're not prepared to change it because you may not want to learn something new or you feel unsure about moving to something new.” (NC05)  “I suppose they just had done it their way for so long, and they felt that what we were proposing was unreasonable, and that if they needed to do a craniectomy, they would still be doing it, whatever the trial showed, because they themselves believed that the operation was beneficial, and so if it's beneficial, they should be doing it.” (NC03)  “You have to deal with existing dogmas, especially in surgery it's still the case that what the boss told you, survives for decades. That's not always critically challenged.” (NC10)  ””I don't want to change the way I've been doing things for years and I've got this, I know this.” I've certainly used this language or I've thought it.” (TC03) |
|  | Interventions that fit established knowledge are more likely to be adopted | NC, IC | “Well, I'm not going to use it unless I have some idea, even though it's wrong, some idea of how it works, I deal with in concussion all the time. So, it's kind of like this thing, if you have to know exactly how it works, you're not going to know exactly how that works.” (NC02)  “…when you're told something, when you have a mechanism that's explained to you as a trainee and you're given an analogy and you're given an explanation that makes sense, about a treatment. And I think this was the case with POLAR, and it appears to be true and then the study doesn't demonstrate that, that's quite a powerful outcome. And, so with POLAR, the whole idea that, you could cool someone's brain down and you don't... I can remember saying to trainees, this is like you've gone and you've sprained your ankle playing sport, whack an ice pack on it.” (IC07)  “It's really interesting from leadership perspectives of how much information does the person on the shop floor need to have. I'll be a little bit medico-centric, but certainly medicos, nursing staff they need to understand the why.” (TC03) |
|  | Simplicity of interventions associated with implementation |  | “…even if I do say, "Okay, this guideline is relevant for my patient." Then there is actually the complexity of the intervention. Is it easy or is it difficult? The reliance on actually does a bedside nurse do it, or does a doctor do it? Because if a bedside nurse can do it, it's probably much more likely to be carefully protocolized and be able to be actioned hour to hour. As we know from many things, if a doctor has to come do it, and things happen on call and at nights, a doctor might not get a bedside for a number of hours and that can influence the lead to adhere to the guideline. I mean, it's well described in many protocols that actually the adherence is much less than would be desired. Even in good units.” (IC04)  “…simple interventions have much more broader ability to be translated because it's so much more approachable. Stuff that can be implemented by junior staff, nursing staff, that's going to be where the money is. And the more complicated TBI interventions are more challenging from that point of view to translate, because it's just, you think about the implementation education resources that are required. Or the effort that's involved to change practice.” (IC01)  “I guess the other major thing is cost. I think that there is a financial implication, a lot of the time with some of the interventions, particularly around some of the newer technologies. So, it is a factor, perhaps not, it's obviously not a priority, but it's certainly is a factor. I think people perhaps require a higher level of evidence to implement new devices or therapies where there is a cost implication.” (IC01)  CONVERSELY, ACCESSIBILITY OF HARMFUL INTERVENTION CAN BE A BARRIER TO EVIDENCE-BASED CARE  “I think something like giving albumin to one of these patients is very easy. Whereas, it's not very easy for a patient to go for a decompressive craniectomy. That's not easy, because the consultant will always be called and there's a few more barriers to that happening.” (IC07) |
| Social/professional role and identity | Different specialists contribute different skills | NC, IC, TC | “Apart from that, in terms of resuscitation fluids, I think as a neurosurgeon we don't do a lot of acute resuscitation unlike the general surgeons so that decision would be left to the critical care doctors or the general surgeons involved with the patients who are resuscitating the patients.” (NC05)  “I certainly wouldn't be comfortable with neurosurgeons telling me how to run the ventilator or what stress ulcer prophylaxis to apply. But our neurosurgeons are very comfortable with that there are certain things that are the province of the intensivist.” (IC03)  “[Neurosurgeons] have very concrete sort of essential interventions, like the placing of EVDs and the evacuating of mass lesions, which I think are essential.” (IC12) |
|  | TBI is not a popular subspeciality among neurosurgeons | NC, IC | “I think having consultant input is probably critical and that is changing over time, but perhaps it could be faster. That probably relates to cultural issue amongst neurosurgical training, that essentially, once you make it to consultant level, you can step away from some of this stuff.” (IC01)  “Traumatic brain injury is not really seen as a route to academic promotion, publications, it's just not seen as a sexy type of thing for a young and upcoming surgeon or young upcoming doctor to follow” (NC05)  “You can count at least in North America, the number of neurosurgeons that are interested in neurotrauma clinically or academically, we're a very small group.” (NC06)  “I have to say though that TBI is not always the favourite of neurosurgeons. So, there are many institutes where neurosurgeons will be perfectly happy to be kept in the background.” (NC10) |
|  | Seniority of decision makers is associated with better practice |  | “Consensus can be reached if productive discussion occurs face-to-face, or at least by telephone between consultants. Where things go wrong, I think, is where neurosurgery registrars are telling senior consultants, maybe in ICU, how to do things.” (NC03) |
| Knowledge | Clinical practice guidelines promote evidence-based practice | NC, IC, TC | “You don't want to make the mistake of making a recommendation that's unique to your organization that isn't represented in any other kind of international guideline or research study. So yeah, I think they have huge part to play.” (IC11)  “[The guidelines are] like a gold standard. They’re by the book but provide a standard from which you can create your own local guidelines flowchart. To deviate from them you need to justify and I find that really helpful.” (IC09)  “Unequivocally, [clinical practice guidelines] make [practice] more evidence aligned… consistent application of a guideline, irrespective of whether it turns out to be good or bad, is likely to be better than a mismatch of heterogonous approaches. Junior doctors, nurses frequently say they like predictability and consistency with respect to how to respond to different clinical and physiological scenarios. And so guidelines give them that. So I guess what I'm saying is a guideline of uncertain validity followed is better than a mismatch of no guideline and a patchwork of different practices often applied inconsistently.” (IC03)  “[Clinical practice guidelines] had a very positive effect in terms of reaching a certain degree of standardization, common recommendations for practice, yes. So, they have probably served to reduce the heterogeneity in practice, which is good.” (NC10)  “I do think guidelines are very important to bring a lowest common denominator up to a certain standard. Guidelines are very good in systems to try and get to a standard of therapy, which is you're trying to uniformize people.” (TC01)  “I like guidelines a lot. And I work for most cases on the ward round, partly for my own benefit just so that I don't forget anything or go way off-piste, but also to show the registrars and nurses that it's okay. If there's a guideline relevant to the patient we're seeing on the ward round, I will always have it up on the computer whenever we're seeing a patient. So whenever we're seeing a TBI patient, I'll have the TBI guideline open on the computer.” (IC06)  “If you're in the middle of nowhere, or you're a busy clinician, and you've got a lot of different things that you have to do, synthesizing the data and putting it into a palatable, usable format for your staff is just a fairly time-consuming activity. If you can then, someone's done that work for you and put it into a nice flow-diagram, which you can copy and paste. I mean, I can just see how that gets widely translated and taken up in lots of different places.” (IC01)  “[Application of the guidelines] actually allows for potential lack of knowledge of certain parties to just accept and use, and by doing that, because the guidelines are usually a systematic review of all available evidence, it's a simplistic way of doing what is acknowledged as current best practice. Then you can circumvent lack of knowledge by doing it in that manner. Okay, so the guidelines are simplistic for the benefit of clinicians that might not want to dig too deeply into the research data themselves. The benefit is that if they were to follow the guidelines, by and large, it is already ratified by the general significant persons in the world with regards to that topic. You are actually most of the time doing best practice in the world. I mean again, you can talk about the level of evidence for best practice but again this is best practice of what we practice in the world. Other countries like Nepal or whatever, they follow it. The assumption is that if we were to follow it, then all outcomes… should be similar.” (NC09)  “We had 22 trauma centres in [region] where we train them up in the guidelines, and we share that the mortality dropped by 45% when the guidelines were put into place. CDC came out with a report, I mean, it's when you do something ridiculous, like use steroids, you under resuscitate, you don't have any brain pressure monitoring, your mortality is close to 50%. And that's what the old days, the 80s, somebody came in with in trauma, 50/50 they'll make it through. Now it's around 20%, 15% mortality, depending upon what the group is, but it's dropped dramatically. And it's dropped because it's just telling people not to do stupid stuff.” (NC02)  “I think that the BTF has been an interesting part of that because they were... I mean, they were the first guidelines created by any surgical specialty. And they fortunately became quickly known as being very high quality, very respected. And I think that's why they've been implemented and forced to a degree that is not seen with other guidelines. And I think part of what's happened with the head injury guidelines, my view, I'd be curious if anyone else has told you the same thing, but I think that clinicians are busy and even if you want to just talk about the big RCTs that are happening in neuro-critical care and in neurotrauma, it's hard for even for the academic folk to keep up with all the trials are coming out… So I think that there's actually a big population of people that says, ‘you know what, I'm going to wait a couple of years till the next edition of the BTF guidelines comes out. They're going to do all the thinking for me, they're going to synthesize.’”(NC06)  “I think guidelines are really important. I'm a big fan of guidelines. I think the Brain Trauma Foundation, even though I think some of the guidelines are shaky, at least they base them on something, and they've been prepared to revise and adjust them sequentially for the last 20-something years. And more than anything, I think they've really helped the management of neurotrauma.” (TC02)  CHALLENGE  “I'm quite sceptical, that writing guidelines changes practice. And I think it's more other things that change behaviours, it's these other things we've talked about. By making it harder to provide an intervention that might not be recommended by the group, I think certainly would be more useful than writing a lot of guidelines that people may not consult.” (IC07)  “…unfortunately the translation of research findings in the guidelines is very haphazard. If you look at the Brain Trauma Foundation, the updated guidelines come out approximately every five years. So that if there's a new finding that just misses the last iteration, it can be years and years and years before it's adopted, and incorporated into practice.” (IC04) |
|  | Adherence to guideline is dependent on quality of underlying evidence | NC, IC | “I think it's got to be pretty robust evidence, like I think our culture is very much around randomized controlled-trials, large sample sizes, plausible effect sizes. Stuff that comes out of left field and things, or just observational data sets, I think people don't respond to that as effectively.” (IC01)  “I think everybody has that experience where you read a guideline and then you actually look up the reference, either discover that the paper that that sentence is based on is very weak or it doesn't seem to have anything relevant.” (IC05)  “The next barrier I guess would be people kind of trusting or accepting [the evidence]. Whether it's just a kind of healthy scepticism for new ideas, just having seen kind of fads come and go, or low quality kind of research, not being substantiated in subsequent bigger trials, sort of physiologically plausible ideas that aren't kind of held up when they're tested in randomized trials and things. So then there may be a just to kind of have a healthy scepticism for jumping on every new kind of idea that comes out, for fear of it not being... Subsequently not being found to be as useful or robust as it was thought to be. And so a part of that is, if you got the information, I guess you got to trust it.” (IC02)  “…we know that most traumatic brain injury guidelines, when you talk about levels of evidence, they're not that high, you see?” (NC09)  “As you are aware, much of the evidence supporting treatments is relatively weak, and that kind of leads to a situation where people feel relatively free to use their own procedures” (NC10)  GOOD QUALITY EVIDENCE SOMETIMES DOESN’T TRANSLATE  “…there are randomized control trials in intensive care that immediately change practice with superb implementation. There are RCTs with low quality evidence that lead to implementation when you probably couldn't justify it based on the quality of evidence. Then there are situations where there are really good RCTs that should lead to change and it's a slow and laborious process, and it's really challenging to understand the behavioral psychology that lies behind that variability.  “The quality of evidence that drives that decision-making is uncertain. Probably in 20 or 30 years' time we'll have a much better idea as to whether following what was in the BTF Guidelines was good or not so good for patients.” (IC03)  “So I think it's in part, it's easy to poke holes, even in the best studies that are getting done. And there's a lot of people that would rather poke the holes than change the practice.” (NC06) |
|  | The current evidence base doesn’t capture the complexity of TBI pathophysiology | NC, IC, TC | “…the barrier of a guideline is it stifles sometimes clinical and individual thinking for customized care on individual patients sometimes. Because it's after all a guideline, it's not a protocol.” (TC01)  “The other problem that has been a feature of research, is everyone wants to look at isolated brain injuries. It's a bit like, “I'm interested in red roses, but I'm only interested in red roses that grow three feet tall, that don't get thrip that have come out of the German synthetic rose factories.” And so I get this information, and then I become a rose garden expert, and I give advice on the 99% of other roses that have got nothing to do with this particular thing, and that's the problem with… our approach to neurotrauma, that the brain injury response is related to other systems, is related to coagulation, it's related to how your blood pressure's maintained, now your heart functions, how your muscles heal, and how we treat people, and so we need to have systems that look at the brain-injured patient who may have multiple injuries, and what's the interplay?” (TC02)  “…unfortunately, management of complex disease states like traumatic brain injuries can be so nuanced. And it's really easy to find a reason to do something different…. It's very rare you have a perfect patient that is described in a perfectly designed trial.” (IC11)  “It's just a multifactorial complex situation, and people don't easily want to believe a particular finding because it's not in their experience to understand the pathophysiology or to expect that that particular treatment modality or change in protocol would actually make a significant difference to their practice or to the outcome of their patients… I think a lot of our work has been extrapolated to the whole field of neurotrauma so that one size fits all, and an operation's been canned; because there are indications where craniectomy is a good thing to do, but people interpret our studies, saying that all craniectomies are now forbidden because we've shown a bad outcome. That's not what we're saying at all. Not what we said in the trial, and we can't extrapolate the findings of the trial to all TBIs. That's the thing that people don't quite understand about these randomised controlled trials, that they don't necessarily apply to all conditions in TBI. The nuances are not understood by the general, certainly, neurosurgical community and maybe, to some degree, intensive care community as well.” (NC03)  “I still have mixed feelings. I'm very supportive of the guidelines, but guidelines are general. Guidelines are based on group evidence, and are not at the current moment, targeted to individual patients.” (NC10)  NC10: “[The evidence base] doesn't really look at the needs of that specific patient who you are treating at that moment. Some patients, just as an example, can have brain swelling primarily due to vasodilatation. If you look at it that way, a mechanical procedure such as decompressive craniectomy may not be effective, however if you have an agent that would limit or reduce the vasodilatation and perhaps barbiturates are an agent that do that, also by decreasing the metabolism, but also by having a direct vasoactive effect. If you would target those patients with vasodilatation with barbiturates, that might be much more effective. AM: So, to me, and I'm taking the example of the decompressive craniectomy because of your center's vast experience there, if you use that mechanical approach in patients with primarily swelling of the vasodilatation, it's doomed to failure. Conversely if you have a patient with a large contusion, and that to me is of particular interest, because over the years of my practice I've seen many patients with hemorrhagic contusions with mass effect where the treating physician decided not to operate. Those patients are the typical example to me of patients with head injury who talk and die. They come in with a relatively good GCS. No-one is really worried, and 24 or 48 hours later, they're dead. So, personally I have become a little bit more aggressive in indications for surgery in those patients. Now, the British School used to say you should never operate on patients with a contusion, because there will be viable neurons within the contused tissue, so don't do surgery. What people have then been doing, is not removing the contusion, but doing a decompressive craniectomy, which to me is not the correct approach if you don't remove the cause of the mass lesion. So, taking those general approaches and applying them without consideration to the underlying pathology, and understanding the pathology better, is not the way we should go in the future.  DG: Do you think that those nuances are adequately captured by the evidence as it currently exists?  NC10: No, they're not.” (NC10)  “I don't know what it is about the head injury field. It could be because it's such a complex pathophysiology. You have such significant heterogeneity. Both in the types of injuries and within patients, you have heterogeneity based on the day from the injury. And it's also not something that you can predict. I moved into vasospasm management in research now, because it's a condition that's predictable. It doesn't happen before day three after subarachnoid hemorrhage. You can, in a way predicted. It's a far more homogeneous pathology as opposed to head injury when you have a patient that has diffuse axonal injury, subdural hematoma, hydrocephalus, concussions. All happening in the same brain. And three days later, other processes set up and you can never predict.” (NC08)  “What I try and teach people is that guidelines are not, as you said, they're not best care for everyone, but they are a benchmark. So when you go to a patient's bedside, you can say, "Okay, well for this patient, that particular guideline isn't right but it's for these reasons." So it's sort of a comparator and I think there's an awful lot of places that don't think that way. It's more of a cookbook.” (NC06)  “I guess that any of us along with any of the surgeons could think that the specific patient’s situation isn't necessarily covered by the evidence generated by the trials. The outcome of the trial applies to a group of people… that would have fitted into the study population, and any particular patient… may actually benefit from individual kind of thinking. I guess that skill [intensivists] and the surgeons bring to the care of individual patients is their problems are kind of unique and don't necessarily need a sort of textbook treatment.” (IC02)  “If you start off with maybe just the relevance for my patient, which would be, okay, so the guideline is for patients with TBI, but actually for X and Y reasons my patient might not fit, the general cohort might be slightly different. So there might be a reason just not to adopt it at all. (IC04)“ |
|  | Guidelines are not pragmatic | NC, IC | “They don't provide data that can, or guidelines that can really be rapidly operationalized. And they don't have the usual tiered therapy. Look, I understand that they perhaps didn't want to provide a recipe for TBI. I understand that they just want it to provide perhaps the principles. But in reality, and come back to an earlier point, if you want to have rapid translation of whatever it is, you need to provide it in a very usable format. Something that can be given to an educated clinician at the bedside and say, "This is how we want you to apply this intervention in a complex situation like TBI." The Brain Trauma Foundation guidelines, I don't think do that.” (IC01)  “The other angle here which I feel is under-appreciated is that guidelines typically start with a review of the literature, which only represents a subset, sometimes a small subset, of the clinical decision-making or the clinical decisions that need to be made for the patient. If there's a clinical decision that has no literature, it frequently is ignored in guidelines.  “…guidelines that are substantially deficient with respect to providing guidance to the full set of clinical decisions are seriously inadequate, in my opinion.” (IC03)  “One of the problems with the guidelines in, say, the fourth edition of the Traumatic Brain Injury Guidelines, which I think the most recent one, from the Brain Trauma Foundation, if you look a those guidelines, they're not very user friendly. They're not easily applicable in the real world, a lot of those guidelines.” (NC03)  “the fourth edition [of the BTF guidelines] of 2016, where if you go through the entire bookwork, which of the recommendations there can you actually apply in practice? It's not very many. To me it gives a good summary of available evidence, but it kind of... forgive me for saying so, but it kind of halts at synthesis of evidence like a systematic review. It doesn't tell you really how to treat TBI or how to treat ICP. Don't give steroids. Okay, that's been generally accepted. So there's no specific problem there of barriers or facilitators. When do you treat ICP? They suddenly come up with 22, which is an artifact of a statistical analysis based on a limited number of patients. If you want to change policy, and people generally would do it at 20 or 25, it makes no sense to suddenly change that to 22 based on limited evidence. So to me that is creating a barrier, just by doing that.  “If you want to really change practice, it has to be meaningful. From a clinical perspective, there's no meaningful difference between 22 and 20. So, if new guidelines change the threshold, but that change lacks clinical relevance, clinicians are not impressed.” (NC10)  “The guidelines are generic, in some ways too methodological.” (NC07)  “I think we're at a point now with guidelines where... So what? We’ve become methodologically perfect, the guidelines are... They're so beautiful, that they're not practical, because we don't have anything new, that's the problem. There's nothing new, and that's where my discussion, maybe after this is going to be about.” (NC02) |
|  | Non-specialist clinicians may lack knowledge | NC, IC | “I think the way our hospital and Intensive Care Unit runs at any moment of the day, there may or may not be at a consultant with an interest in the management of the particular problem present at the bedside. So the chance of something being applied or implemented that our so called experts, might see as undesirable is significant because I think the time before the message gets out that something may or may not be an appropriate intervention, it has to disseminate amongst the whole consultant group of say 15 consultants. And then the senior registrar group who are going to be recommending interventions to the bedside, and then nurses who also need to understand that perhaps, they have the right to the question intervention that might be requested by well-intentioned registrar. So the albumin example: I think that can easily happen because all it requires is a register who's rotating from perhaps anaesthetics, or someone who asks you, or even someone who has not come across that before, and that happens quite easily because the volume of ICU is enormous. It's very easy for someone not to have read the SAFE study if they started training five years ago, because it's now becoming ancient literature and unless someone refers them to it, they may not be aware of it. So I think it's the barriers to implementation, I suppose, the breadth of ICU, still the relative infrequency of these patients in our unit, even though we have one or two of these patients at any time, there's a large number of consultants, doctors in training and nurses. So, for all of them to be aware of what the data might show and then interpreting it in the same way, is the next thing. I think that really takes a lot of time. So, for me, the barriers are, I suppose, the nature of our workforce. The fact that no one can be aware of all the evidence, even major evidence in areas in which they don't work all the time.” (IC07)  “I think the main barrier is the fact that in a lot of hospitals we don't have designated neuro ICU. We usually have to work in the framework of a generalist ICU. And then trying to introduce new techniques or even implement new ways of looking at all data can be difficult when you don't have the pool of people who have the interest the knowledge and a subspecialty interest to drive this forward. I mean, we're trying to look at the Bonanza study and how brain oxygen monitors can help us manage these patients. But a lot of the times, there are even problems with the basic management of patients in ICU, in terms of just simply ICP and CPP control. And depending on who’s on and their experience, sometimes you'll have trouble implementing even the basic recommendations by the Brain Trauma Foundation. And I think the main problem is the fact that this neuro ICU concept has not really taken off at least here in [region]. I've experience working in the neuro ICU in [region] at the [Hospital]. And my experience there was very favourable, very positive.  “I think it's the fact that the staff have a better grasp of the topic. I mean, you can't really expect the generalist to be having the same in-depth knowledge as a subspecialist in any topic.  “It's not as simple as just sticking the monitors in a patient. It's what it actually means in terms of how that changes management, how are those numbers incorporate into the overall picture. And you can't do that with someone who doesn't understand the basics. You have to have people who understand, for example, the Brain Trauma Foundation guidelines. Why their guidelines actually recommend, who understand the research behind them.” (NC08) |
|  | Education and training facilitate evidence-based practice | NC, IC | “…education, obviously. So having a strong culture of things like journal club that incorporate to making sure that registrar teaching is delivered with the latest knowledge and research, or knowledge from research findings in mind. I think there's always a gap in bringing consultants up to date. In every specialty there are consultants, I'm sure, who's in depth level of knowledge on many topics stopped the moment they passed their exam. And most of us will try to stay up to date, but I certainly know that... I mean you can't stay across everything. And working in an environment like we do where we rotate through different pods, there are areas of our practice at the Alfred that I don't find as interesting as other areas, so it will be more difficult to stay up to date. So I do think that is a ... the process that we use to educate registrars, journal club, teaching so on…” (IC06)  “Facilitators can be lots of things like journal clubs and research groups where research findings are discussed. They are honed to local practice, guidelines are developed. So those things are facilitators. I think scientific meetings are very good and where research is discussed, I mean the critical care reviews and stuff where we we talk about a number of our trials, I think it's particularly good. The new findings are presented, they're editorialized, they're critiqued, they're available in podcasts and they're available for junior mid-career researchers to assess information in bite sized chunks on the way to work as well. So you don't have to be a dedicated researcher to get access to information.” (IC04)  “I think things, that have helped us as a group with the questions like decompression and temperature, so hypothermia in these patients, is we've actually over the last couple of years done this in journal club more than once. We've done it with our registrars, we've done it with a lot of the consultant group more than once. And we've done it with the neurosurgeons in the room. And quite a few of them have attended on one occasion or another. And I think that's helped to stop people perhaps asking for therapy that they thought might be beneficial, just based on the fact they weren't exposed to the evidence.” (IC07)  “I think it certainly comes into medical student training and into resident training. I think the colleges have a responsibility to include a very strong element of research training in the curriculum. The Neurosurgical Society has tried to do that. They introduced a compulsory period of research during training, but it's ... and they have to publish at least one article, I think, before they sit their exam and things like this that they've ... But these are ... they're not token efforts, but they're actually probably relatively small element of the overall training package. Maybe it has to be that way because there's so much other stuff they have to learn, but there are ways of putting the research components into all aspects of their learning, so that they actually have to look at the evidence base for particular elements in neurosurgery, neurosurgical practice, which they do. Which they do ... The good trainees will know the literature, but the weaker trainees will not. Some of them still ... They can still manage to pass the exam, but they don't have a great understanding or feeling for the evidence base. You can't force it, but at least the college, the neurosurgical training board is aware of this issue. I think they do seriously attempt to improve the understanding of the trainees, or the evidence base, or knowledge of the trainees in the evidence base or neurosurgical practice.” (NC03)  MORE THAN FORMAL EDUCATION, INFORMAL INTERACTIONS DRIVE PRACTICE  DG: “How effective do you think strategies of education, journal club, informal conversations between consultants and guidelines, how effective do you think they are in ensuring alignment between evidence and practice?”  IC06: “I think probably not very. If I were to rank them in their effectiveness I'd probably say informal conversations and then guidelines and then journal club. I think the personal connection of me bumping into a researcher or a more experienced colleague, or even a colleague who's more interested in a particular area than I am, this is just talking in general terms, and them saying, "Oh, you know." Me saying, "How would you do this?" And then them saying, "Oh, this is ..." (IC06) |
|  | Awareness of current evidence-practice gaps is important | NC, IC, TC | “The first thing we did was we did a survey, it's funny how things repeat over the lifetime, so we did a survey, we got a nurse and we paid her to call every trauma centre in the United States, and speak to the head nurse in the ICU to figure out what the practice was. We had a list of questions, and it turned out that steroids are rampant, everybody's getting steroids, everybody is under resuscitating patients. Only 20 or 30% of patients were getting monitored, it was terrible.” (NC02)  “The thing about the U.S. [military healthcare] system that I really admired was that they were able to collect data, clinical data, as they changed their practices. Everything was evidence-based, and everything they did was based on outcomes. They had a very quick feedback loop.” (NC03)  “So I think there's real barriers around our willingness to be agnostic to the quality of the care we deliver and to be open to delivering better care. I really see that repeatedly as a big problem. We just constantly think we already deliver the best care, so what's the point?... How well do we really know what we deliver? You know? You remember, years ago, we did that audit of albumin in TBI at [Hospital]. And I expected to find definitely no patients who received albumin. And yet we found something like 5%, maybe 10% of our TBI patients. Yeah, like that's shocking.” (IC08)  “In the trauma audit role…I try and bring in information and see what's next, perhaps. Have certainly raised those points as options to think about to push. As we all know directors at times will make decisions to say, hey, this is important at the moment, or no, this at the moment, isn't… I'm a big believer in those numbers. It just gives objectivity and it gives the ability to question in I hope a safe way.” (TC03) |

TDF = Theoretical Domains Framework

## Table S2: Factors perceived to influence practice in fluid resuscitation of patients with severe traumatic brain injury

| TDF Domain | Themes | Specialists | Illustrative quotations |
| --- | --- | --- | --- |
| Environmental context and resources | Policy environment / availability of intervention | NC, IC | “Any hypoosmolar fluid, it's like you need nuclear codes to get access to those.” (NC06)  “The most effective facilitator of the implementation of that particular piece of evidence was now a requirement… where the blood bank will only release albumin when there's a consent to the administration of blood products… I think SAFE-TBI was very poorly applied in new patients with TBI. Really, what changed the albumin use in TBI has probably been the policy change with respect to consent.” (IC03)  “There are many different drivers, so there can be things that are historical practice. There can be the availability of fluids in areas where these patients are cared for. Sometimes people just pick things off the shelf.” (IC04)  “It is probably something that is a practice that could be changed just by making one fluid or another fluid less available.” (IC06)  “The reality is that albumin in our unit does require a little bit of extra effort, you have to order from the blood bank, takes some time to come. I think the lack of ease of access probably stops misadventure in that sense…  [Overseas] albumin was expensive and due to cost more than evidence albumin was not used in these patients. It was a cost argument, because clearly albumin doesn't cost us money. Whereas for them, it costs them $100 or $200… I actually think they never used albumin in these patients because of the cost.” (IC07)  “We don't give much colloid, never give albumin… As far as I know it's only ever been available from the blood bank.” (IC10)  “Fluid availability and ease of access… if it's absolutely imperative that a traumatic brain injury patient does not receive a certain fluid type and it keeps occurring in an institution, then you have to remove that fluid type as an option.” (IC11) |
| Beliefs about consequences | Physiological targets | NC, IC | “In neurosurgery we tend to have a very pro sodium approach to our fluid choice. And that's not just for the traumatic head injury. I mean we drive into the juniors and are always checking sodium and their fellows to take place.” (NC01)  “I think it’s going to come down to just a normal physiology.” (NC04)  “Sodium osmolality, pH all factor into my decision-making.” (NC06)  “The other thing I see influencing the choice is physiological arguments. So I think when patients are very shocked, I think people extrapolate and say, oh, gosh, I've already had a bit of fluid. I've given a little bit of this other fluid, I've given some crystalloid and they're still shocked. I've seen people give colloid in this case.” (IC07)  “But so many other factors come into it… The composition of the fluid itself, what they understand, of the basic science. It's not like those things are not important, they are all important, but I guess we all pick from all the possible inputs, what we believe to be the most important, and those vary between us… I told my trainees, “ICU is about learning physiology so that you know it, so that you can then disregard it. You're not allowed to disregard it until you know it, and once you know it, you must disregard it.”” (IC08)  “I don't think we should be using hypotonic solutions, and there's probably not, robust, randomized controlled-trial data to support that. But I think that's a biologically plausible stance to take, position to take. So question is, should we use Plasma-Lyte or should we use Hartmann's or should we use saline? Now, the concern there is obviously the chloride component and the chloride loading and the potential risk for acute kidney injury.” (IC01)  “From a scientific thinking point of view, the choice of fluid should be based on… the best evidence we've got, improving mortality or functional outcomes.” (IC03) |
|  | Competing priorities of different injuries | IC, TC | “It may be influenced by what the other injuries are.” (IC03)  “They've got the competing interests of the pelvic fracture and the spleen, and what does that need? But what are the potential adverse effects of those units of blood to what we're doing oxygen-wise or whatever else with our blood and brain and blood brain barrier and unknowns as well. I guess having seen that degree of evolution in so many of our practices, it also remains for me what we can't measure. We don't quite know yet.” (TC03) |
| Social influences | Key opinion leaders | NC, TC | “The easiest to respond is, [Local Champion] is my intensivist… if the case becomes complicated, then we call for [Local Champion] and his guys. It's easy for me.” (NC07)  “Therefore if we go back to facilitators for change, it's again, almost in a simplistic way that peer group pressure, isn't it. Getting this information from people like [Local Champion], huge mentor early on used to be well, change practice. I certainly haven't prescribed albumin.” (TC03) |
| Knowledge | Inconsistent awareness of evidence | NC, IC, TC | “I'll be honest with you, all the time that I was in Utah, I don't think we ever talked about that study.” (NC06)  “I think that there was a study from long time ago, I have to look it up, that looked at colloid versus crystalloid.” (NC09)  “I am not aware of clear recommendations on the normal fluid management of a TBI patient.” (NC10)  “I use normal saline for volume in traumatic brain injury patients and only normal saline, because I am aware of other fluids having been shown to be detrimental. Exactly which trials I'm now talking about, I don't really know.” (IC12)  “I'm fairly fluid agnostic. I do not think there's any strong evidence in the majority of patients for one choice of fluid over another.” (IC06)  “I think some of the things that influences a doctor's choice of fluid in this patient group would be how much Intensive Care they've done, their direct exposure to... whether they've actually read the relevant articles pertaining to this.” (IC07)  “It's not that those findings had no effect, they clearly do, they are important findings and they... It's hard to know for sure, but I think it certainly contributed to a decrease in use. It's just that it should have been immediately zero, like boom gone, that's it. But so many other factors kind of come into it.” (IC08)  DG: “How do you apply the results of the SAFE-TBI Study in your practice?  IC11: “It's the fluid one?... I probably don't put as much weight on it as a lot of my colleagues.” (IC11)  DG: “What about albumin? You know the SAFE study? How do you apply that in your practice?”  TC02: “I don't really, but I don't mind if it's used.” (TC02) |
|  | Awareness of publication has changed practice | IC | “I never give them albumin because there’s evidence showing that’s potentially harmful” (IC09)  “I don't think there's a role for anything else than normal saline in TBI patients. Certainly, the SAFE TBI sub-study suggested harm associated with 4% Albumin in resuscitation of TBI patients.” (IC01)  “My experience is that ICU registrars and senior registrar, particularly ICU senior registrars and consultants would shy away from giving 4% Albumin in these patients… So has it changed practice? I think it has.” (IC07)  “Clearly the SAFE trial on traumatic brain injury related to albumin, conclusively proved that that is pretty bad. I think that is level one evidence there. I haven't seen any evidence out there to contradict that, and that's my personal opinion. So that's a great study. My clinical decision making has been influenced based on that.” (TC01) |

TDF = Theoretical Domains Framework

## Table S3: Factors perceived to influence practice of surgical decompressive craniectomy in patients with severe traumatic brain injury

| TDF Domain | Themes | Specialists | Illustrative quotations |
| --- | --- | --- | --- |
| Beliefs about consequences | Decompressive craniectomy makes ICP easier to manage | NC, IC | “The ideal patient in my books is someone that has an underlying good brain, they're just declining because of high ICP. So trying to make sure that it's a patient that has a good brain, that just needs a lower ICP.” (N06)  “We're trying to treat acute physiology in the hope that we will actually improve long-term mortality and outcomes.” (IC04) |
|  | Management of ICP not shown to improve outcomes | NC | “So, in the case of a patient with refractory ICP, I would generally say that that is a very serious problem which we have tried to treat, but have not been successful yet. That there is the possibility to do a decompressive craniectomy. That that would be effective in reducing pressure, but the big risk would be, to keep someone alive in a severely disabled state. That message put that way, is of course linked to your estimate of prognosis.” (NC10) |
|  | DC improves survival but not functional outcomes | NC, IC | “I think that yes, it probably reduces death, but probably increases vegetative outcome is the way I sort of put the papers together.” (NC06)  “The main thing I think in TBI is who should we go all the way and who should we let go, because that is the gold standard. Again, that is what we want to know, prognostication for the patient and who to push and who not to push… the main thing here is how do we design a research trial to show function.” (NC09)  “DECRA has quite clearly indicated that wide bifrontal decompressive craniectomy as an early tier intervention for patients with raised ICP is harmful. So then you really just considering its role as a rescue intervention in patients with refractory ICPs.” (IC01)  “For diffuse TBI, my understanding of the evidence is that it doesn’t improve outcomes. It might improve your ICP, it might improve how long you stay alive for Ut with worse functional outcomes.” (IC09) |
|  | DC should only be done after maximal medical therapies have failed | NC | “I think we all accept that it's a second tier therapy, which should only be considered when pretty much every other option has failed.” (NC08)  “The DECRA trial shows that we probably, if possible with diffuse cerebral oedema, definitely go with thiopentone and barbiturate coma first for sure because I think that's the important thing, okay? That one we agree wholeheartedly, the ICU management, no difference. Before this, we would say let's take the bone off and then barbiturate later, right?” (NC09) |
|  | Increased intensity of ICU therapies including barbiturates means less need for DC | NC | “The key point is whenever we really [intensively medically manage] in ICU our patients, the number of decompression is very small, is reduced to a minimum… If you see the study, the study is based on an ICP persistently over 25. Our intensivist don't leave us much cases with ICP persistently over 25.” (NC07) |
|  | Age of patient associated with outcomes | NC, IC | “in general, it's something that can be considered in young patients” (NC08)  “If it's an older patient, who's got a lot of co-morbidity then it's often something that's not really an appropriate intervention. Because I think that if you do want to take it, then you're likely to be left with a very disabled survivor.” (IC01)  “I would think the patients they would have excluded would have been younger than 18 and older than 70, or something like that. So I suppose we don't look after kids and if I wouldn't recommend it in a 50 year old or a 30 year old who has probably more recoverability, I certainly wouldn't recommend it in 75 or 80 year old.” (IC07) |
| Environmental context and resources | Cultural context and resources change practice (also maps to Social influences) | NC, TC | “In certain societies, like in China and in parts of India and Southeast Asia where it's expected that if the patient comes in with a severe head injury, that surgery is part of the management, because surgery saves lives. Therefore, we have to put these patients through surgery, and there is no ICP monitoring in many of these parts of the world.” (NC03)  “The Congo situation, or Tanzania, or Vietnam, or Cambodia I receive a patient, the CT scan is bad. The patient is something in between moderate and even mild, but the CT scan is bad. I have either small or no access to ICU, no monitoring. In some countries, no repeat CT scan, because it's too expensive. So what I do, I decompress the patient, because I know that these days is the cheapest way to control ICP. Which we call it the prophylactic decompression, which we don't see…  “We are in a Catholic culture, so only God can take off your life. This is our culture. So it's difficult to discuss with family because whenever you say I have 1% of possibility, they want 1%. But if you discuss with families about the fact that this is a procedure is life saving, but you may have a severe disability and even the vegetative state as outcome. And we are confident that it may happen in a high percentage of cases. Then I think we have changed, any times we do it, whenever we can together with the intensivist, we tend to inform the families. And we have seen more and more that some families will tell you, "No, I don't think. When he was alive he didn't wanted this. So we say, no." (NC07)  “The second thing is going to be decided by the cultural experience. For example, you've seen that variations from an Anglo-Saxon family to someone else is going to be significantly different.” (TC02) |
| Social influences |  |  |  |
|  | Decisions should incorporate family’s input on patient’s values | NC, IC, TC | “Traditionally, we might've approached, the patient's got, your brother or mother or whatever's got high pressures, we need to take the bone off. You need to sign here. These are the risks. Now it's much more, these are our options. This is the way we're thinking. I would recommend this or recommend not doing this.” (NC01)  “The big problem I've always found is that patients get wheeled into clinics, three years afterwards, say, "Thanks a lot for all of that, but no one mentioned this was on the cards. No one said anything about this. It was all about life and death." And that's where we've really failed. So now we can be far more clear about that, saying, "There is a real chance he could be vegetative or severely disabled. He may get used to that, he may be perfectly happy." But as you know as well as I do, there are lots of people who really don't do that well and their lives are pretty wrecked after it” (NC04)  “That becomes a decision that is based on a number of factors, the individual patient, or family's expectations about their healthcare outcome, the age of the patient, their ability to undergo rehabilitation and indeed the nature, I guess, of the underlying brain injury.” (IC01)  “Of course the family has a role in sort of informing the process, but never in deciding an operation in the sense of, should it be done or not. Only in the sense of the values of the patient and whether what we're proposing meets those values. Is it in line with those values or not? So yes, if it's like, is this operation in terms of what we believe the outcomes achievable? Is that in line with the values, as you understand them of your loved one.” (IC08)  “I think communicating with the family has been really helpful because I think they're more fearful of severe disability than death.” (IC11)  “I believe the family should be involved from the beginning itself to know the express wishes of the patient. Even if it's a young patient. Who knows, they might've told about some express wish before and to understand in a certain that patient's wishes and the ability of the family to be engaged in their loved one.” (TC01) |
|  | Cultural context and resources change practice | NC, TC | “In certain societies, like in China and in parts of India and Southeast Asia where it's expected that if the patient comes in with a severe head injury, that surgery is part of the management, because surgery saves lives. Therefore, we have to put these patients through surgery, and there is no ICP monitoring in many of these parts of the world.” (NC03)  “We are in a Catholic culture, so only God can take off your life. This is our culture. So it's difficult to discuss with family because whenever you say I have 1% of possibility, they want 1%. But if you discuss with families about the fact that this is a procedure is life saving, but you may have a severe disability and even the vegetative state as outcome. And we are confident that it may happen in a high percentage of cases. Then I think we have changed, any times we do it, whenever we can together with the intensivist, we tend to inform the families. And we have seen more and more that some families will tell you, "No, I don't think. When he was alive he didn't wanted this. So we say, no." (NC07)  “The second thing is going to be decided by the cultural experience. For example, you've seen that. Variations from an Anglo-Saxon family to someone else is going to be significantly different.” (TC02 |
| Emotion | Internal/external pressure towards active intervention | NC, IC, TC | “It's just really hard if you're looking at a patient and a family and you know that if you don't do the decompression, the patient probably going to die, it's very hard not to do it even if you know that DECRA is suggesting there's going to be a bad outcome. It's hard withhold life-saving care, and I think that motivates an awful lot of us.” (NC06)  “The one major thing about neurosurgery is if you're not sure, you do something about it. That is why sometimes we operate on cases that are futile.” (NC09)  “If you refuse to do surgery where the family wants to take every single opportunity for improvement, and the patient dies, the surgeon is to blame, and the family cannot accept the death. Their entire mourning process is disrupted. If on the other hand, you do do the surgery as they wish, and the patient does not survive, they will accept it.” (NC10)  “So many of our treatment decisions are based around our emotional responses to the patient and their unwellness, not necessarily the patient. If you've got a patient with high ICPs, you emotionally feel bad… when neurosurgeons walk in and they look at an ICP chart, they have an emotional response to that, and that's driven by, I think, the natural bias that all clinicians, not just surgeons, have to do something when doing nothing should be regarded as a legitimate option.” (IC03)  “But for this patient, he's a young man. He has a family. Let's do it. We need to do it… there'll be that ethical moment of we know this probably won't make a difference, but we will want to do as much as we can for this person.” (TC03) |
|  | Family may struggle to understand complexity in pressured situation | NC, IC | “Families will often view coming in for decompressive craniectomy as the best thing where it'd probably save the person's life but not their function.” (NC05)  “The family opinion is important, but I also am skeptical about how well we are able to convey the complexity of it to a family, usually under a moderately time-pressured situation when they're under extreme pressure.” (IC12) |
| Social/professional role and identity | Management of ICP deferred to ICU |  | “We've gone from a very active approach, to much more passive one, without being disrespectful to the medical management.” (N01)  “In Europe, it's the intensivists who are the decision makers. They have the primary responsibility for the care… The primary responsibility for the decision making remains with the intensivist.” (NC10) |
|  | Seniority of decision making is important | IC | “A lot of the decision-making, some of the decision-making, quite a bit of the decision-making, is registrar driven. If you get a registrar who says, "Ah, I'm thinking about doing a decompressive craniectomy," the intensivists might say, "Oh, that'd be a good one to discuss with the neurosurgical consultant."” (IC03)  “I think to circumvent those problems, clear communication with the neurosurgeon specialist has been how I've tried to get out of those problems. The issue being they're so busy than registrars and they're offered a lot of independence when it comes to ward based management, that I think there's a lack of communication up their hierarchy.” (IC11) |
|  | Collaborative, interdisciplinary decisions at a senior level and including families enable patient-centric decisions | NC, IC, TC | “It's a team event really. You've got to be on the same page. And you've got to value their opinion, and you've got to agree which is the way forward. And that's got to be done early on really. And as I'm sure you find yourself, not all neurosurgeons are particularly easy to work with, same as intensivists, it's got to be a two way thing. But you do have to come through it together. And these are consultant to consultant conversations.” (NC04)  “If there's going to be a discussion with a family about consent for decompressive craniectomy, there'll usually be a consensus or at least a tacit consensus with the neurosurgeons from the ICU staff. Then there'll be typically a discussion between the neurosurgeons and the family regarding consent for that procedure.” (IC03) |
| Knowledge | Publication has changed practice | NC, IC, TC | “Going through both DECRA and RESCUEicp, and looking at the long term outcomes, and seeing some of my patients long term after immediate decompressive Craniectomy, I really put a brake on it. I'm not as enthusiastic about it anymore, and in fact, I think there may come a day where there would be very few people having it.” (NC02)  “I think the DECRA trial has very much affected my own personal practice in that I would very rarely perform that unless asked to perform it by one of the critical care doctors; and I would probably look at the patient and say well is it a young patient, where are their bifrontal contusions, is there a mass legion, what else is going on with the patient? And basically apply the DECRA criteria to that patient and then I would probably go ahead and do that.” (NC05)  DG: “Would you say that DECRA and RESCUE have changed your practice?”  NC07: “Not that much, because we were already in the same track.” (NC07)  “[The trials] have led, I would say in the western world, to a decreased number of decompressive craniectomies.” (NC10)  “I think that definitely influenced my personal practice and I think they've definitely influenced Australian practice. I know the North American practice was very much towards decompressive craniectomy, and I know that the British or UK practice still, because of the results of RESCUEicp, they continue to use decompressive craniectomy as a rescue therapy. But I think in Australia, that you would find that it's a relatively rare procedure.” (IC01)  “My experience subsequently [following publication], over the last few years, that it's very rarely discussed, very rarely done.” (IC02)  “The results of DECRA and RESCUEicp, would drive that. So I would have been a strong advocate of bifrontal decompressive craniectomy, patients with refractory ICP. I would do that less now, but however, still in some targeted situations, in consultation with the neurosurgeons, we would do a decompressive craniectomy.” (IC04)  DG: “Do you think that the results of DECRA and the RESCUEicp trial have changed practice?”  IC08: “No, as far as I can tell at [Hospital], no. I think it gives the surgeons a nice out when they don't want to operate and they ignore it when they do, but I don't think it's really changed a thing.” (IC08)  “That has changed since the DECRA trial, in that our unit in general was pretty quick to go to decompression in the past. Whereas now, unless there is an expanding hematoma or something where it is very obviously indicated, it's now further down our protocol.” (IC10) |
|  | Repeated findings more powerful | IC | “My understanding… was that DECRA probably didn't change practice a great deal because people who wanted to do it would still do it, and people that didn't want to do it still wouldn't. But I think RESCUEicp probably has changed practice a bit more in that the people who were on the fence have now moved towards not to do [decompressive craniectomy]. And that's, I think, both neurosurgeons and intensivists. And I remember having a meeting with us and the neurosurgeons, not all of them, when we were discussing the trial. And this seemed to be a general consensus amongst the neurosurgeons in keeping with our view that this would be a treatment for exceptional circumstances rather than something that we routinely offer.” (IC06) |
|  | Trial results are difficult to reconcile | NC | “I too have lost a lot of sleep trying to interpret DECRA and RESCUEicp as I think everyone else has.” (NC06) |
|  | Injury appearances on CT are key to deciding whether to perform DC | NC, IC, TC | “If the patient has got widespread brain injury, particularly generalized swelling with significant Diffuse Axonal injury. Again, I think that you will likely generate a disabled survivor. If they have a very focal area, focal mass lesion, or a focal area of swelling, then I think that becomes a more nuanced decision.” (IC01)  “There's the main question I suppose, is whether or not the patient has a diffuse or localized injury and whether or not the results of DECRA apply to this patient.” (IC12) |
|  | Trials don’t capture complexity of TBI pathophysiology | NC, IC, TC | “What it boils down to, in my way of thinking is that, despite all efforts to do the best trials possible, they just don't capture the subtle differences between patients.” (NC10)  “Our practice is probably a point along that ordinal scale that was not tested in DECRA and for which we don't know if it is better or worse than the two points in DECRA that were tested.” (IC03) |

TDF = Theoretical Domains Framework
